# Supplementary material for: Single-Mode Laser in the Telecom Range by Deterministic Amplification of the Topological Interface Mode
Source: ACS Photonics. 2024 Feb 12;11(3):1006–11. doi: 10.1021/acsphotonics.3c01372 (PMC10958602; doi:10.1021/acsphotonics.3c01372)
Supplement: Supplementary file 1 — ph3c01372_si_001.pdf [file ph3c01372_si_001.pdf]

Supplementary information

Single mode laser in the telecom range by deterministic amplification of the topological interface mode

Markus Scherrer[1], Chang-Won Lee[2], Heinz Schmid[1], Kirsten E. Moselund[3,4,*]

[1] Science of Quantum & Information Technology, IBM Research Europe – Zurich, 8803 Rüschlikon, Switzerland

[2] Institute of Advanced Optics and Photonics, Hanbat National University, 34158 Daejeon, South Korea

[3] Laboratory of Nano and Quantum Technologies (LNQ), Paul Scherrer Institut (PSI), 5232 Villigen, Switzerland

[4] Integrated Nanoscale Photonics and Optoelectronics Laboratory (INPhO), EPFL, 1015 Lausanne, Switzerland

[*] Correspondence: kirsten.moselund@psi.ch, +41 56 310 34 15

Comparison between topological interface and bulk modes

With fig. S1, we want to emphasize the difference in electromagnetic field intensity distribution between the topological interface (TI) mode and the 'trivial' photonic crystal modes. This is key to our design concept as it enables the deterministic amplification of the desired topological interface mode. Fig. S1 a gives a cross section of the local field intensity through the center of the device, where the confinement in the one dimensional array leads to the strongest value. The positions of the two unit cell sites *A* and *B* are highlighted in different color at the top and bottom, allowing to directly relate intensity and position.

For the TI mode, intensity maxima fall exactly on the positions of the *A* sublattice and the minima overlap with the *B* sublattice. For the two trivial modes, the intensity is more evenly distributed within both sublattices. Note that the mode with a higher frequency has its highest intensity in the space between two nanorods – it belongs to the so-called 'air band', whereas the lower frequency side of the photonic band gap makes up the 'material band'.

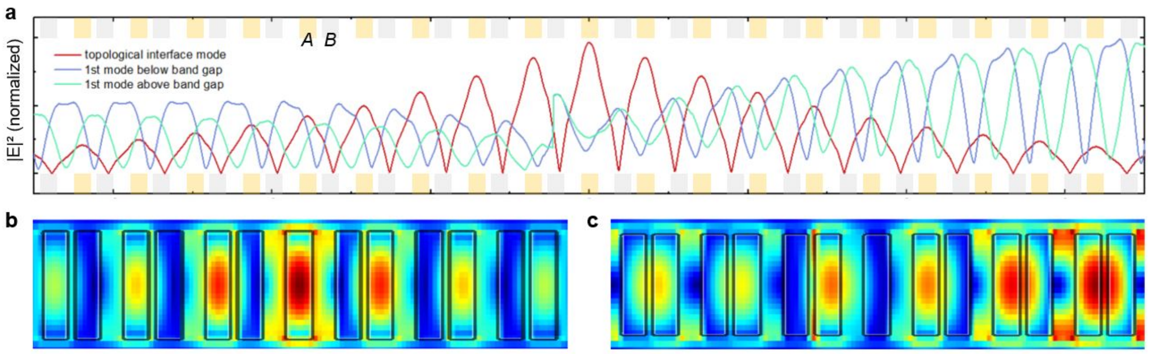

Fig. S1 Electromagnetic field distribution within the topological photonic nanorod lattice. a Cross-sections of the field intensity for the topological interface mode (red) and the first trivial modes for both below (green) and above (blue) the photonic bandgap. Relating the maximum (minimum) of the topological interface mode intensity reveals the high overlap to the sublattice *A* (*B*) marked by yellow (grey) color. **b, c** Top-view mode profile in the device center for the topological interface and a trivial mode, respectively.

The overlap fraction of each mode with the respective unit cell sites is given in table S1, again highlighting the difference of the TI mode for the two sublattice sites. Its intensity is almost four times higher on the *A* sublattice, whereas the trivial modes in fact have a slightly higher intensity on the *B* sublattice.

Tab. S1 Calculated modal overlap of the two sublattice sites. The significant difference in distribution is obvious for the topological interface mode. Numbers in percentage, the remainder is distributed in the oxide cladding in between the nanorods.

	**Sublattice *A***	**Sublattice *B***
**Interface mode**	47.7	12.5
**'Air' mode**	27.9	34.9
**'Material' mode**	31.8	37.2
